# Preclinical study of a novel therapeutic vaccine for recurrent respiratory papillomatosis

**DOI:** 10.1038/s41541-021-00348-x

**Published:** 2021-06-18

**Authors:** Maxwell Y. Lee, Simon Metenou, Douglas E. Brough, Helen Sabzevari, Ke Bai, Caroline Jochems, Jeffrey Schlom, Clint T. Allen

**Affiliations:** 1 Section on Translational Tumor Immunology, National Institute on Deafness and Other Communication Disorders (NIDCD), National Institutes of Health, Bethesda, MD USA; 2 Precigen, Inc., Germantown, MD USA; 3 Laboratory of Tumor Immunology and Biology, National Cancer Institute, National Institutes of Health, Bethesda, MD USA

**Keywords:** DNA vaccines, Viral infection

## Abstract

Activation of antigen-specific T-lymphocyte responses may be needed to cure disorders caused by chronic infection with low-risk human papillomavirus (lrHPV). Safe and effective adjuvant therapies for such disorders are needed. The safety and efficacy of a novel gorilla adenovirus vaccine expressing a protein designed to elicit immune responses directed against HPV6 and HPV11, PRGN-2012, was studied using in vitro stimulation of T lymphocytes from patients with recurrent respiratory papillomatosis, in vivo vaccination studies, and therapeutic studies in mice bearing tumors expressing lrHPV antigen. PRGN-2012 treatment induces lrHPV antigen-specific responses in patient T lymphocytes. Vaccination of wild-type mice induces E6-specific T-lymphocyte responses without toxicity. In vivo therapeutic vaccination of mice bearing established HPV6 E6 expressing tumors results in HPV6 E6-specific CD8+ T-lymphocyte immunity of sufficient magnitude to induce tumor growth delay. The clinical study of PRGN-2012 in patients with disorders caused by chronic infection with lrHPV is warranted.

## Introduction

Chronic infection with low-risk human papillomavirus (lrHPV) type 6 or 11 can cause neoplastic disorders such as recurrent respiratory papillomatosis (RRP)^1^. Induction of a lrHPV antigen-specific T-lymphocyte response is likely necessary to clear a chronic lrHPV infection. Although numerous immunotherapies designed to induce HPV16-specific T-lymphocyte immune response have been developed for cancer^2–4^, few such immunotherapies have been developed for lrHPV-driven disorders^5,6^. Therapeutic vaccines designed to induce antigen-specific T-lymphocyte responses in the setting of an existing HPV16 infection have demonstrated clinical activity through the induction of HPV-specific T-lymphocyte immunity^3,4^. Similar therapeutic vaccines may be effective for disorders caused by infection with lrHPV. Therefore, the treatment of patients with RRP caused by lrHPV types 6 or 11 with a therapeutic vaccine may provide benefit.

Here, we describe the preclinical development, safety, and efficacy of a novel therapeutic vaccine, PRGN-2012. This vaccine induced lrHPV antigen-specific responses in peripheral T lymphocytes from patients with RRP and in vaccinated mice. PRGN-2012 treatment in mice bearing established lrHPV antigen-positive tumors also generated a lrHPV antigen-specific CD8+ T-lymphocyte response sufficient to allow tumor trafficking and delayed tumor progression. These data provide the preclinical rationale for the clinical study of PRGN-2012 in patients with RRP.

## Results

### PRGN-2012 induced HPV-specific responses in T lymphocytes from patients with RRP

**Fig. 1:**
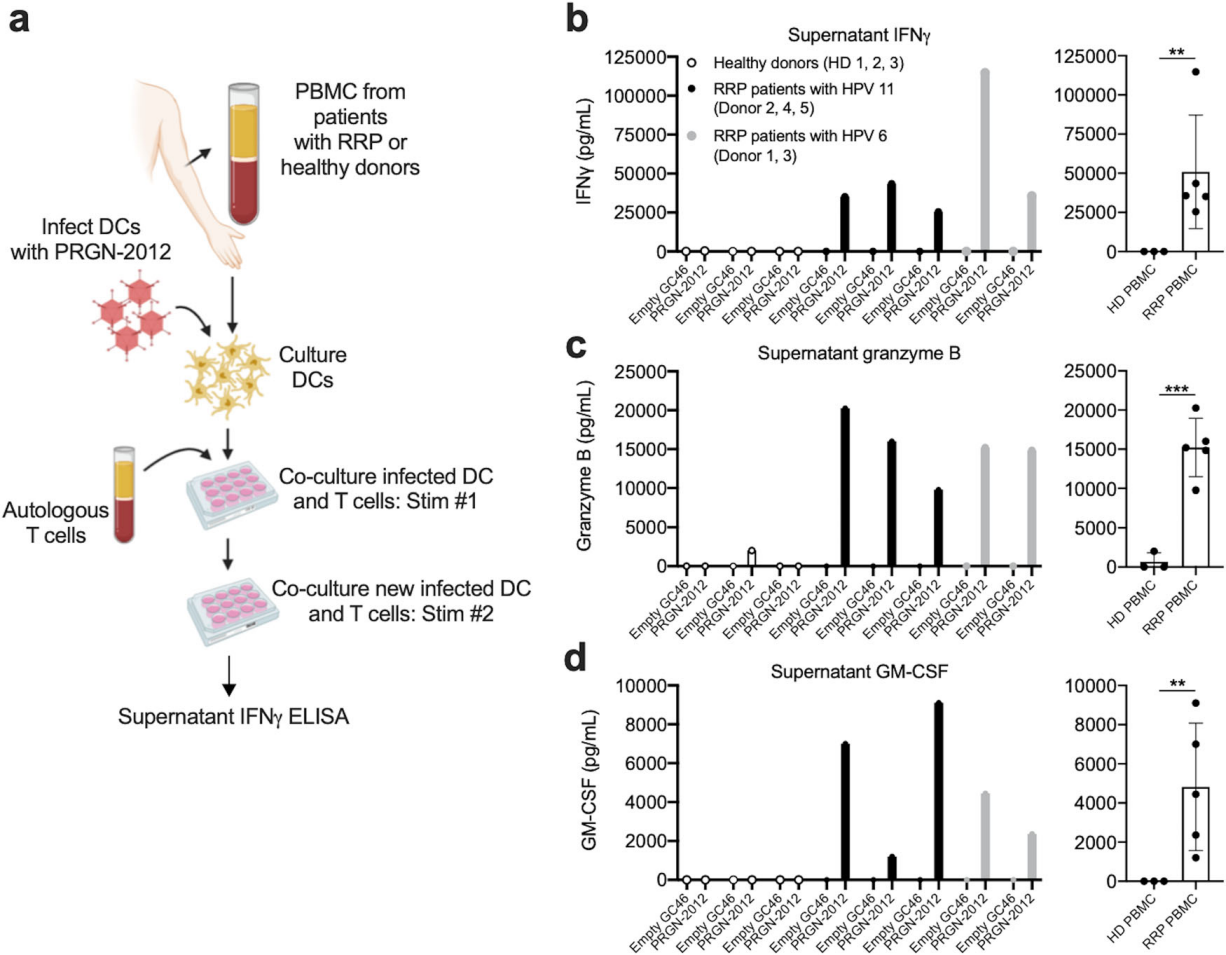
In vitro stimulation with PRGN-2012 induced HPV antigen-specific responses in T cells from RRP patients. **a** Experimental design of in vitro stimulation of T cells isolated from the blood of patients with HPV6 or HPV11-driven RRP or healthy donors with PRGN-2012. Concentrations of **b** IFNγ, **c** granzyme B, and **d** GM-CSF in the supernatant of cocultures assessing HPV antigen-specific responses in T lymphocytes isolated from the blood of patients with HPV11-driven RRP (*n* = 3, black bars), HPV6-driven RRP (*n* = 2, gray bars), or healthy donors (*n* = 3, open bars) following two rounds of in vitro simulation with PRGN-2012 or empty GC46. Displayed results are the result of HPV antigen-specific stimulation after baseline cytokine concentrations following in vitro stimulation with empty GC46 were subtracted. Similar results were observed in two independent assays. PBMC peripheral blood mononuclear cells, DC dendritic cells, HD healthy donor, empty GC46 GC46 gorilla adenovirus without HPV gene inserts (control virus). **p < 0.01; ***p < 0.001; Student’s *t* test.

Study of clinical specimens collected from patients enrolled in previous clinical trials for RRP demonstrated few active papilloma-infiltrating T lymphocytes specific for HPV6 or 11^7^. To address this deficit in HPV antigen-specific T lymphocytes, PRGN-2012 and controls were assessed for their ability to induce HPV antigen-specific responses in peripheral T lymphocytes from patients with HPV6- or 11-driven RRP or healthy donors (Fig. 1a). In vitro stimulation (IVS) with PRGN-2012 induced HPV-specific responses in the form of IFNγ (Fig. 1b), granzyme B (Fig. 1c), and GM-CSF (Fig. 1d) production and release from T lymphocytes isolated from five patients with HPV6- or 11-driven RRP with diverse HLA class I haplotypes (Supplementary Table I) but not healthy donors. Flow cytometric analysis revealed that IFNγ was produced by CD4+ and/or CD8+ T lymphocytes in a donor-dependent fashion following IVS with PRGN-2012 (Supplementary Fig. 1). IVS with empty GC46 lacking HPV DNA induced IFNγ, granzyme B, and GM-CSF to a lesser degree (Supplementary Fig. 2). These results demonstrated that PRGN-2012 could induce baseline inflammation and HPV antigen-specific activation of T lymphocytes derived from the peripheral blood of patients with HPV6- or HPV11-associated RRP.

### In vivo vaccination with PRGN-2012 was safe and induced E6-specific T lymphocytes

**Fig. 2:**
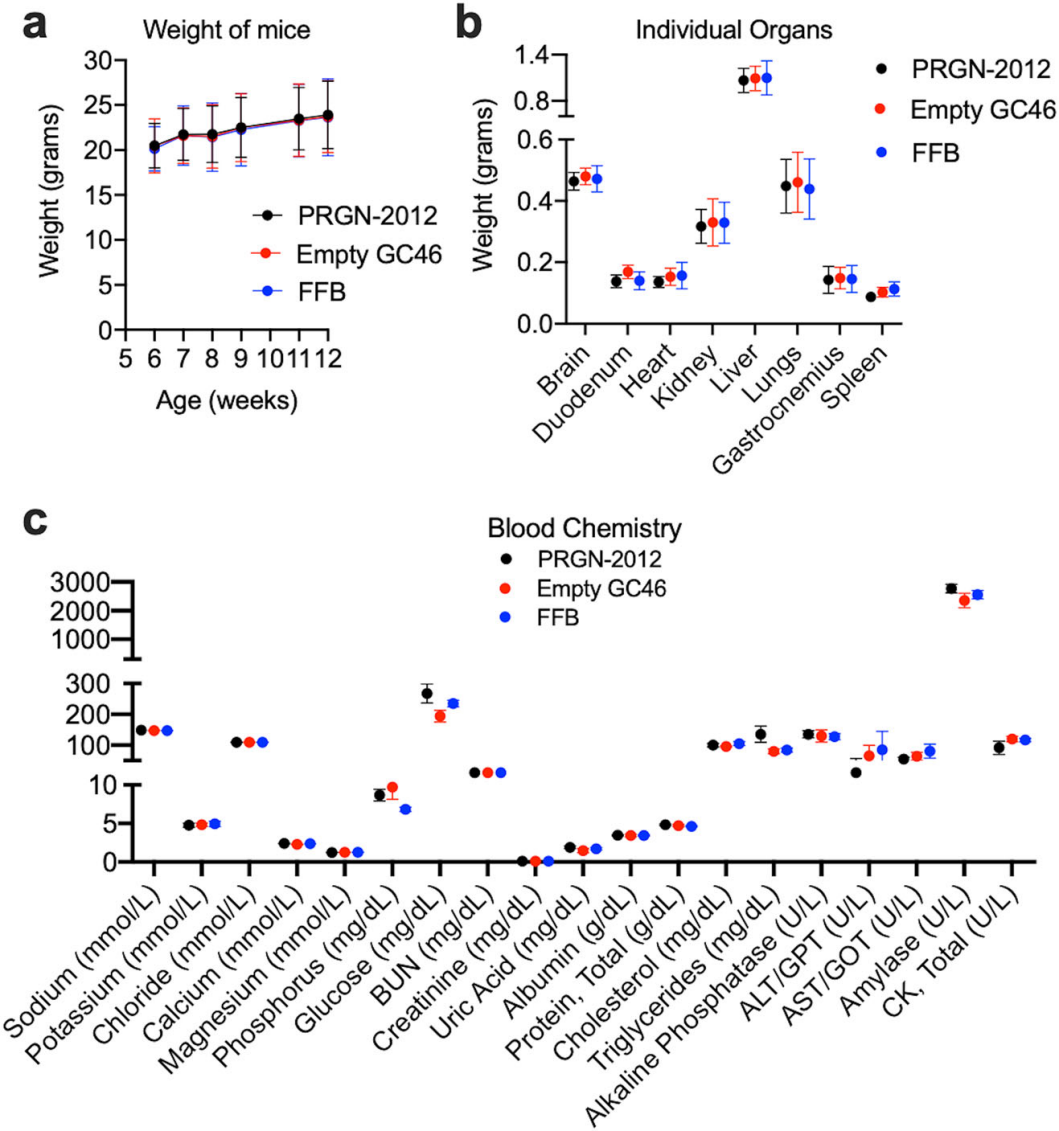
No toxicity was observed following in vivo vaccination with PRGN-2012. Wild-type C57BL/6 mice were vaccinated with three doses of PRGN-2012 (10^10^ VP), empty GC46 (10^10^ VP), or FFB. **a** Body weight was assessed serially in vaccinated mice (*n* = 5 male and 5 female mice/group). 24 h after the final vaccination with PRGN-2012, empty GC46, or FFB, **b** organs were harvested and weighed and **c** plasma/serum was collected for chemistries (*n* = 5 male and 5 female mice/group). For chemistries, units of reported measurements are listed for each test on the *x*-axis. FFB final formulation buffer.

To first assess the safety of PRGN-2012, wild-type (immunocompetent) C57BL/6 mice were vaccinated via a homologous boost and prime regimen of subcutaneous injections and assessed for evidence of toxicity. Vaccination with PRGN-2012 did not induce weight loss in the mice (Fig. 2a), did not alter the weight of any major organ (Fig. 2b), and did not significantly alter plasma or serum chemistry measurements compared to treatment with empty CG46 or final formulation buffer (Fig. 2c).

**Fig. 3:**
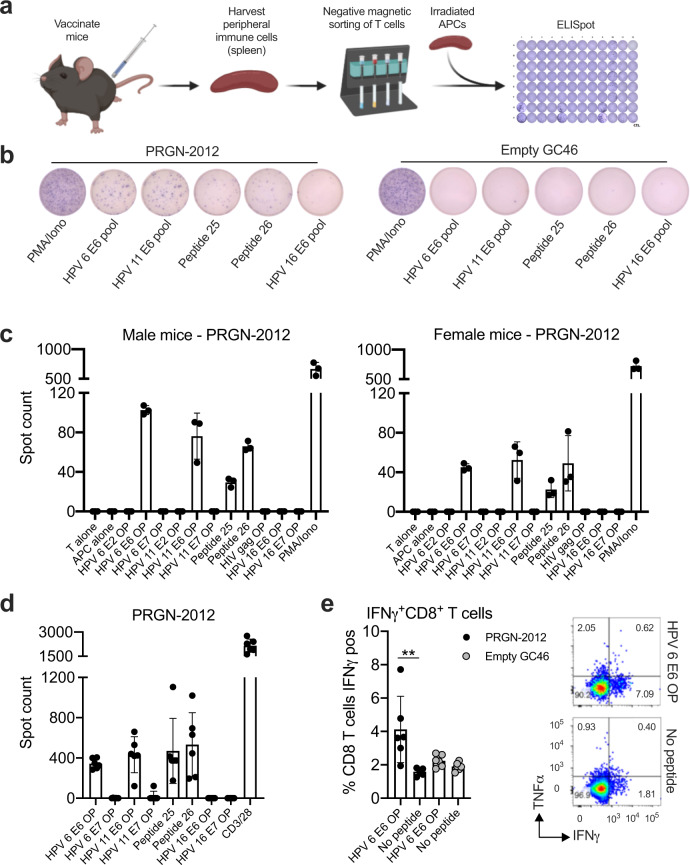
In vivo vaccination with PRGN-2012 induced immunity against an E6 epitope shared between HPV6 and HPV11. **a** Experimental design of peripheral T lymphocytes from wild-type C57BL/6 mice vaccinated with PRGN-2012 assessed for HPV antigen-specific immune responses. **b** Photomicrographs of representative ELISpot wells demonstrating responses to HPV6 and 11 overlapping 15-mer peptide pools as well as synthesized minimal peptides following in vivo vaccination with PRGN-2012 but not empty GC46. **c** Quantification of IFNγ spots in vaccinated male (*n* = 3) and female (*n* = 3) mice. **d** Quantification of IFNγ spots in an independent validation in vivo vaccination experiment in female wild-type C57BL/6 mice (*n* = 5). **e** Quantification (left) and representative flow cytometry dot plots (right) of CD8+ T-lymphocyte IFNγ production following assessment of HPV antigen-specific T-lymphocyte responses from mice vaccinated with PRGN-2012 or empty GC46, measured by intracellular flow cytometry (*n* = 6/group). Dot plots show gated live, CD3+CD8+ T lymphocytes from mice vaccinated with PRGN-2012. Similar experimental results were observed in three independent experiments. APC antigen presenting cell, OP overlapping peptides. **p < 0.01; Student’s *t* test.

To assess for the ability of PRGN-2012 to induce an immune response to HPV6 or 11, C57BL/6 mice were vaccinated and splenic T lymphocytes were assessed for HPV6 or 11 E2, E6, or E7 antigen-specific responses (Fig. 3a). T-lymphocyte responses to 15-mer overlapping peptides covering each HPV protein individually or to minimal epitopes predicted to be high affinity MHC class I binders via in silico study were assessed with coculture ELISpot assays. In vivo vaccination with PRGN-2012 but not empty GC46 induced T-lymphocyte responses against 15-mer overlapping peptides corresponding to HPV6 E6 and HPV11 E6 in both male and female mice, suggesting the presence of one or more shared epitopes (Fig. 3b, c, expanded data in Supplementary Fig. 3a, b).

Stimulation of these T lymphocytes with individual 15-mer peptides revealed positive cytokine (IFNγ) responses to two peptides (peptide 25—TTAEIYSYAYKHLK and peptide 26—IYSYAYKHLKVLFR), suggesting the presence of T lymphocytes specific for a minimal epitope derived from the larger peptides. In silico study of predicted binding to murine MHC class I molecules revealed 8-mer peptides with very low IC_50_ scores predictive of binding to H-2K^b^ derived from HPV6 E6 (E6_44-51_; YSYAYKHL; IC_50_ 3.5) and HPV11 E6 (E6_44-51_; YAYAYKNL; IC_50_ 4.6). These data suggested a degree of promiscuity in H-2K^b^ binding of this semiconserved E6_44-51_ epitope between HPV6 and 11. This experimental result demonstrating induction of E6-specific T-lymphocyte cytokine responses (IFNγ, with additional cytokines MIP-1α and RANTES) following in vivo vaccination with PRGN-2012 but not empty GC46 was replicated in a larger independent cohort of female C57BL/6 mice (Fig. 3d, Supplementary Figs. 3c and 4). Flow cytometric analysis revealed greater production of IFNγ by CD8+ T lymphocytes upon exposure to HPV6 E6 15-mer overlapping peptide following vaccination with PRGN-2012 compared to empty GC46 (Fig. 3e). Taken together, these data demonstrated that in vivo vaccination with PRGN-2012 can prime and activate CD8+ T lymphocytes specific for one or more epitopes present within both HPV6 E6 and HPV11 E6.

### PRGN-2012 treatment induced CD8-dependent HPV-specific immunity

**Fig. 4:**
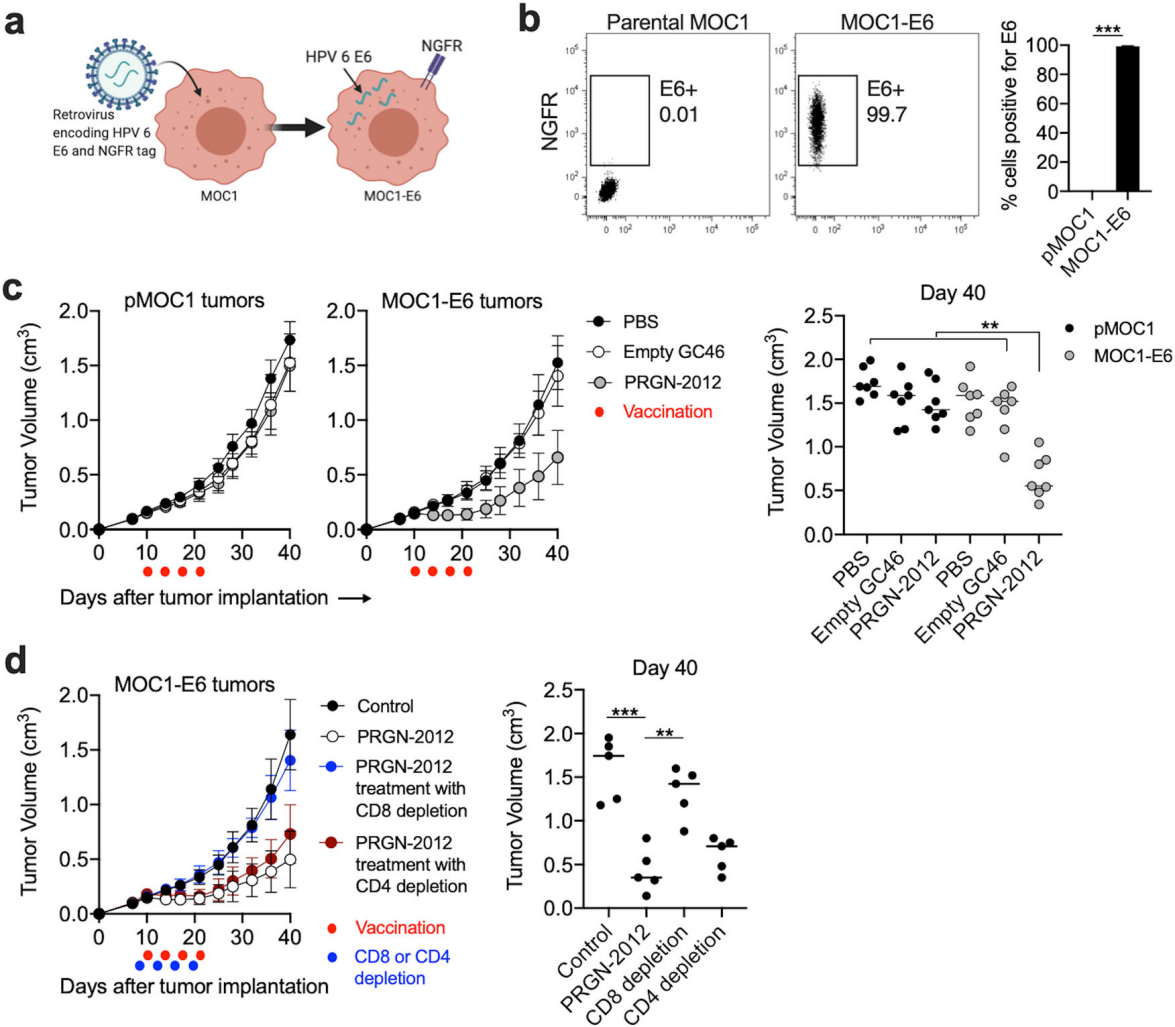
PRGN-2012 induced tumor growth inhibition of carcinomas engineered to express HPV6 E6. **a** Diagram of the retroviral transduction to create MOC1 cells that express HPV6 E6. **b** Flow cytometry dot plots demonstrating E6 (NGFR) positivity in parental MOC1 or MOC1-E6 cells, quantified on the right. **c** Summary tumor growth curves of mice bearing parental MOC1 or MOC1-E6 tumors (*n* = 7 mice/group) treated with PRGN-2012, empty C46 or control (PBS). Red dots indicated treatments. Day 40 tumor volumes are individually quantified on the right. **d** Summary growth curves of mice bearing MOC1-E6 tumors treated with PRGN-2012 in the presence or absence of CD8 or CD4 depleting antibodies. Red dots indicate treatments and blue dots indicate depletions. Day 40 tumor volumes are individually quantified on the right. Similar results were observed in two independent experiments. NGFR nerve growth factor receptor. **p < 0.01; ***p < 0.001; Student’s *t* test or ANOVA.

It was unclear if induction of peripheral HPV antigen-specific T lymphocytes would be sufficient to control the growth of a neoplasm harboring cells expressing HPV antigens. Given the lack of reproducible in vivo murine models of low-risk HPV-driven neoplastic diseases, a transplantable syngeneic mouse model of head and neck cancer (mouse oral cancer 1; MOC1) was engineered to express HPV6 E6 (Fig. 4a) with high purity (Fig. 4b). Therapeutic vaccination of C57BL/6 mice bearing established MOC1-E6 tumors resulted in tumor growth delay (TGD) (Fig. 4c). Therapeutic vaccination with PRGN-2012 had no effect on mice bearing parental MOC1 tumors lacking HPV6 E6. This experiment was repeated in the presence or absence of CD8 or CD4 depleting antibodies to establish which cell type was responsible for the observed TGD. Systemic depletion of CD8 but not CD4 T lymphocytes with monoclonal antibodies abrogated the TGD observed with PRGN-2012 treatment of MOC1-E6 tumors (Fig. 4d). These data established that therapeutic vaccination of mice bearing established E6 antigen-positive tumors with PRGN-2012 can induce CD8-dependent immune responses sufficient to inhibit tumor growth.

### PRGN-2012 treatment induced tumor infiltration of effector T lymphocytes specific for E6

**Fig. 5:**
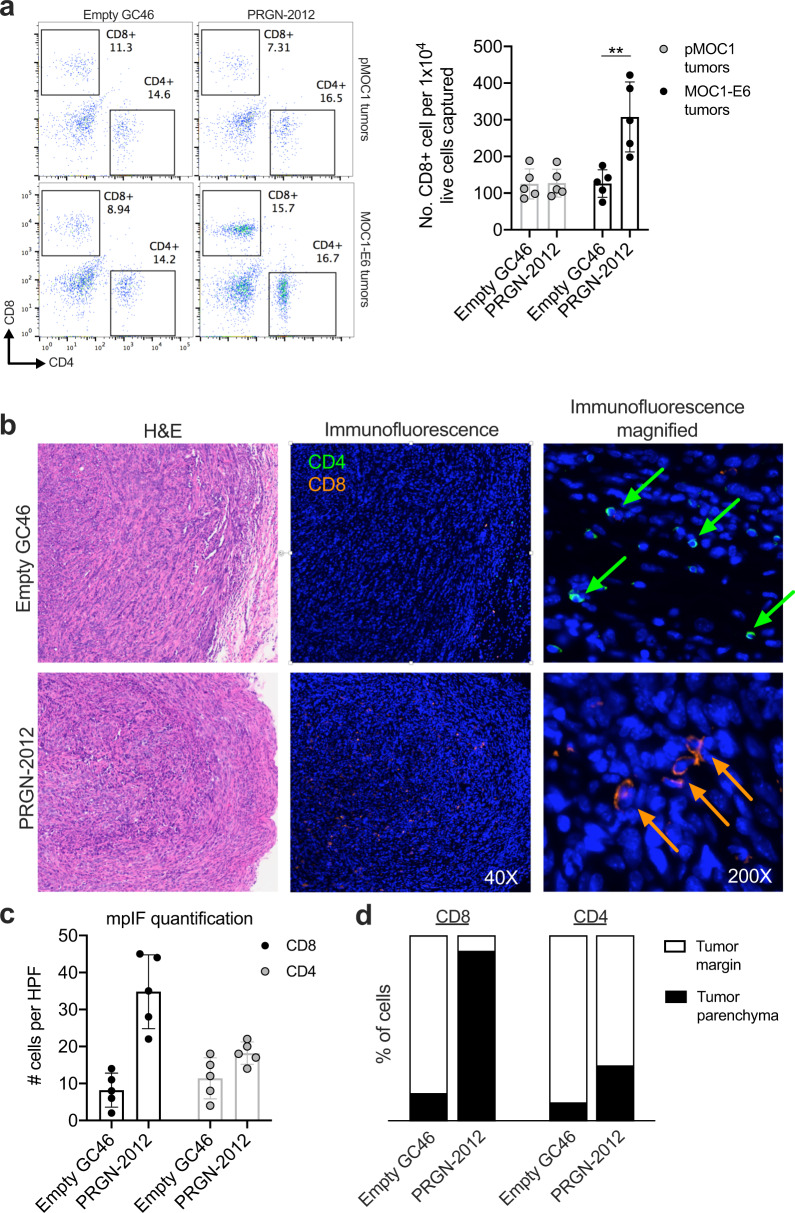
PRGN-2012 promoted trafficking of CD8+ T lymphocytes into the tumor parenchyma. **a** Representative dot plots of freshly digested parental pMOC1 or MOC1-E6 tumors obtained from mice treated with PRGN-2012 or empty GC46 assessed for T-lymphocyte accumulation via flow cytometry. Normalized quantification is shown on the right. **b** Representative H&E and immunofluorescence photomicrographs demonstrating localization of T lymphocytes within MOC1-E6 tumors treated with PRGN-2012 or empty GC46. **c** Quantification of total T lymphocytes per high power field (HPF). mpIF multiplex immunofluorescence. **d** Quantification of tumor margin vs tumor parenchyma localization of CD8+ and CD4+ T lymphocytes.

Flow cytometry of tumors harvested from parental MOC1 or MOC1-E6 tumors treated with PRGN-2012 or empty GC46 was used to determine changes in T-lymphocyte infiltration. Significantly greater numbers of CD8+ T lymphocytes were present in MOC1-E6 tumors treated with PRGN-2012, but not in parental MOC1 tumors treated with PRGN-2012 or MOC1-E6 tumors treated with empty GC46 (Fig. 5a). Immunofluorescence was next used to determine the spatial localization of these T lymphocytes. Whereas analysis of MOC1-E6 tumors treated with empty GC46 revealed CD8+ T lymphocytes localized to the periphery of the tumor, MOC1-E6 tumors treated with PRGN-2012 demonstrated increased numbers of CD8+ T lymphocytes that infiltrated throughout the tumor parenchyma (Fig. 5b–d). PRGN-2012 treatment did not significantly increase CD4+ T lymphocytes.

**Fig. 6:**
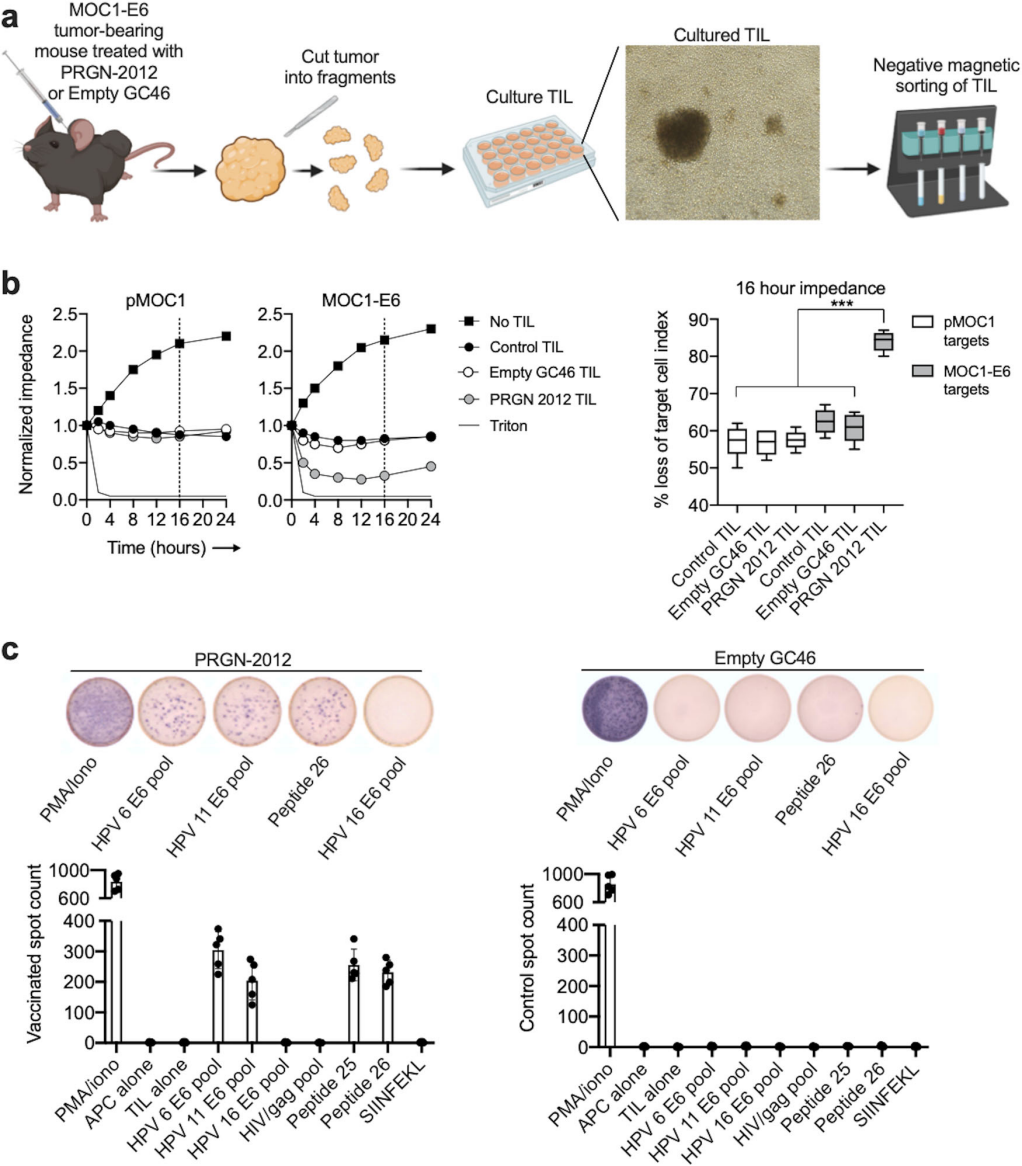
HPV antigen-specific effector T lymphocytes infiltrated tumors following PRGN-2012 treatment. **a** Schematic of TIL cultured from tumors from MOC1-E6 tumor-bearing mice treated with PRGN-2012 or empty GC46 vector (*n* = 5 mice/group). **b** Representative impedance analysis of cultured TIL from MOC1-E6 tumors treated with PRGN-2012, empty GC46, or control (PBS), then cocultured with either parental MOC1 or MOC1-E6 target tumor cells (*n* = 5 tumors per condition). TIL are added to target cells at experimental time 0. Percent killing (represented as % loss of target cell index) is quantified 16 h after the addition of TIL to targets and is shown on the right. **c** Photomicrographs of representative ELISpot wells and quantification of IFNγ spots demonstrating responses to HPV6 and 11 overlapping 15-mer peptide pools as well as synthesized minimal peptides in TIL cultures from MOC1-E6 tumors treated with PRGN-2012 or empty GC46. TIL tumor-infiltrating lymphocytes. ***p < 0.001; ANOVA.

To experimentally determine if these T lymphocytes measured by flow cytometry and immunofluorescence within MOC1-E6 tumors represent E6-specific T lymphocytes, tumor-infiltrating lymphocyte (TIL) were cultured from tumors from mice treated with PRGN-2012 or empty GC46 and tested for their ability to kill MOC1 cells expressing or lacking HPV6 E6 (Fig. 6a). TIL cultured from mice bearing MOC1-E6 tumors treated with PBS alone (control) or empty GC46 killed MOC1 cells expressing or lacking HPV6 E6 to a similar degree (Fig. 6b), suggesting the presence of TIL specific for endogenous MOC1 antigens. However, TIL cultured from MOC1-E6 tumors treated with PRGN-2012 killed MOC1 cells expressing HPV6 E6 to a significantly greater degree than MOC1 cells lacking E6, suggesting the presence of E6-specific TIL. To validate the presence of E6-specific TIL, coculture ELISpot experiments were performed. TIL cultured from MOC1-E6 tumors treated with PRGN-2012 but not empty GC46 demonstrated responses to 15-mer overlapping peptides corresponding to HPV6 E6 and HPV11 E6 as well as the peptides 25 and 26 (Fig. 6c), similar to the in vivo vaccination studies. These data demonstrated that PRGN-2012 can induce HPV-specific T lymphocytes that can traffic into E6 antigen-expressing neoplasms and induce effector responses sufficient to result in TGD.

## Discussion

Current treatment options for disorders caused by chronic infection with lrHPV cannot reliably eradicate associated disease^8,9^. A curative treatment likely necessitates immunological clearance of lrHPV infection, implying that cytotoxic T cell mediated immune responses targeting cells infected with lrHPV are required. Here, we describe the design and preclinical testing of PRGN-2012, a therapeutic lrHPV vaccine designed to prime and/or reactivate cytotoxic T-cell responses against lrHPV antigens with the goal of eradicating lrHPV infection and associated papillomas. We demonstrated that vaccination with PRGN-2012 elicits lrHPV-specific T-cell responses in vitro and in vivo. Further, therapeutic vaccination induced migration of lrHPV-specific CD8+ T lymphocytes into tumor parenchyma. Although tumors were not cured, therapeutic vaccination delayed tumor growth in this syngeneic model of a neoplasm expressing HPV6 E6 antigen.

The rationale behind therapeutic vaccination is the delivery of an immunogenic payload (nucleic acids or proteins) to antigen presenting cells for processing and presentation of antigens to T lymphocytes in the setting of preexisting infection. Subsequent T-lymphocyte priming and activation may then induce clearance of lrHPV infection and disease^10^. Clearance of an existing intracellular infection such as HPV is unlikely to occur with preventative vaccination which induces a primarily humoral immune response^11^. Previous studies have demonstrated the clinical benefit of therapeutic vaccines for lrHPV disease using the MMR vaccine^6^ and modified vaccinia Ankara encoding bovine papillomavirus E2^5^. It is unclear whether the observed efficacy of these vaccines was due to nonspecific, type I interferon mediated activation of innate immunity given that both of these treatments were administered into lesions directly, and because neither study demonstrated the induction of a specific T-lymphocyte response against lrHPV antigen. A more recent study of an HPV6 E6/E7 DNA plasmid-based immunotherapy demonstrated the induction of an HPV6-specific T-lymphocyte response in addition to clinical efficacy in a cohort of three RRP patients^12^. Together, these studies provide foundational evidence for therapeutic vaccination as a treatment for disease caused by chronic infection with lrHPV. These approaches may be limited by the lack of high-level induction of specific T cells which reduces the possibility of long-term memory T-cell responses. This limitation may be overcome with the use of the GC46 viral platform that has the ability to generate high-level antigen-specific T-cell responses following multiple administrations.

RRP is caused by HPV6 or 11^7,13^. PRGN-2012 is designed to encode segments of both HPV6 and HPV11. T lymphocytes from three patients with HPV11 and two patients with HPV6 associated RRP with diverse HLA haplotypes were activated by PRGN-2012, and peripheral T lymphocytes generated in vaccinated mice and tumor-infiltrating T lymphocytes isolated from tumor-bearing mice treated with therapeutic vaccination responded to peptides derived from both HPV6 and 11. There are likely shared or similar epitopes between closely related genotypes HPV6 and HPV11. Although our experimental design did not exclude T-lymphocyte responses to other epitopes, the most probable shared minimal epitope from HPV6 and 11 as predicted by in silico study was E6_44-51_ restricted to H-2K^b^. The HPV11 E6_44-51_ epitope differs from HPV6 at residues 2 and 7, but also has very high predicted binding affinity to H-2K^b^. These differential positions are not anchor residues for H-2K^b^ binding; position 5 is known as the central anchor “C” pocket and preferentially accepts phenylalanine or tyrosine, which is present in both peptides^14^. Thus, PRGN-2012 is very likely to induce both HPV6- and 11-specific T-lymphocyte responses.

Our IVS of peripheral blood mononuclear cells (PBMC) from RRP patients demonstrated nonspecific inflammation with empty GC46 treatment alone, as well as additional antigen-specific inflammation with PRGN-2012 treatment. However, induction of lrHPV antigen-specific T-lymphocyte immunity in vaccinated mice developed with PRGN-2012 but not empty GC46. In addition, CD8-dependent tumor growth inhibition in MOC1-E6 tumor-bearing mice occurred following treatment with PRGN-2012 but not empty GC46. These data suggest that although the GC46 vector alone is capable of inducing baseline inflammation, the lrHPV payload is necessary for the development of systemic antigen-dependent immunity required for therapeutic TGD. These results support extensive existing data indicating that antigen-specific T lymphocytes are required for therapeutic effects in established neoplastic disease^15^.

Limitations to our study exist. We did not identify minimal epitopes targeted by T lymphocytes following PRGN-2012 treatment in our IVS of human blood products. Dissecting lrHPV early antigen-specific responses could yield important insights into the ability of PRGN-2012 to induce polyclonal T-lymphocyte responses, and this should be performed in prospective clinical trials. We also did not demonstrate responses against other lrHPV early proteins in vaccinated WT mice or MOC1-E6 tumor-bearing mice treated with therapeutic vaccination. C57BL/6 mice only express H-2K^b^ and H-2D^b^ MHC class I molecules, which limits the possible repertoire of class I presented epitopes. Study of blood or tumor-infiltrating T lymphocytes from patients treated with PRGN-2012 may result in the identification of minimal epitopes derived from multiple lrHPV early genes and their associated HLA class I restriction elements.

Effective clinical adjuvants aimed at inducing immunological clearance of lrHPV and cure of RRP are needed. PRGN-2012 demonstrated no evidence of toxicity in preclinical studies and induced lrHPV antigen-specific CD8+ T-lymphocyte responses of sufficient magnitude to infiltrate antigen-positive neoplasms and induce TGD. In addition, PRGN-2012 appeared to induce T-lymphocyte responses against epitopes derived from both HPV6 and 11. Clinical study of PRGN-2012 in patients with papillomatous disease caused by chronic infection with lrHPV is warranted.

## Methods

### Clinical samples and approvals

Healthy donor PBMC were obtained commercially. RRP patient PBMCs were collected under NIH IRB approved protocols NCT02859454 and NCT03707587 with informed consent. Wild-type C57BL/6 mice were purchased from Taconic and all in vivo experiments were approved under an NIH Animal Care and Use Committee approved protocol.

### Vaccine

PRGN-2012 is built on Precigen’s gorilla adenovector platform. The GC46 gorilla adenovector was identified and isolated from nonhuman primate sources^16^, and multiple genes have been deleted to prevent viral replication^17^. The protein antigen expressed from PRGN-2012 is a Precigen proprietary design and is a fusion of regions from human papillomavirus proteins selected by bioinformatics approaches and protein engineering to elicit immune responses directed against HPV infected cells. Final formulation buffer and GC46 lacking HPV DNA payload (Empty GC46 vector) were used as experimental negative controls.

### In vitro stimulation

Dendritic cells from PBMCs were transduced with experimental or control vaccine constructs at a multiplicity of infection of 5000 viral particles/cell for 3 h at 37 °C. Autologous magnetically isolated T lymphocytes (Stemcell EasySep) or whole PBMC were added to the culture (10:1) for IVS. At one or more timepoints following three IVS, supernatants were analyzed for cytokine concentrations.

### Cytokine analysis

IFN-γ enzyme-linked immunosorbent assay or the Milliplex Map Human CD8+ T Cell Magnetic Bead Panel was used per manufacturer’s recommendations. Sample data were acquired on a Luminex Bio-Plex 200 (Bio-Rad).

### Flow cytometry

Human T lymphocytes or PBMC were exposed to GolgiStop/Plug (Thermo Fisher Scientific) for 12 h prior to preparation usingthe Intracellular Fixation and Permeabilization Kit per manufacturer recommendations (Thermo Fisher Scientific). For other experiments, tumors were harvested from mice bearing parental MOC1 or MOC1-E6 tumors 48 h after the second vaccination and processed into single-cell suspensions as described^18^. Anti-mouse CD45 (clone), CD4 (GK1.5), and CD8 (53–6.7) antibodies and anti-human CD4 (RPA-T4), CD8 (SK1), and IFNγ (4S.B3) conjugated antibodies were applied for 45 min. Dead cells were excluded using Sytox (Thermo Fisher Scientific) viability dyes. Analyses were performed on a BD Fortessa running FACSDiva software and interpreted using FlowJo (vX10.0.7r2).

### In vivo vaccination

For toxicity studies, mice were vaccinated with 10^10^ viral particles subcutaneously (flank) on days 0, 7, and 14. For vaccination studies, mice were vaccinated with 10^9^ viral particles subcutaneously (flank) on days 0 and 7. Day 14 splenic T lymphocytes were sorted via negative magnetic selection (Stemcell EasySep Mouse T Cell Isolation Kit). Splenocytes from nonvaccinated mice were pulsed with 1 μM 15-mer peptides (overlapping by 11 amino acids) spanning entire HPV6 or 11 E2, E6, and E7 proteins or minimal epitope (8–11 mer) peptides predicted to be high affinity binders to H-2K^b^ or H-2D^b^ via in silico prediction (IEDB) or control peptides. Peptide pulsed, irradiated (18 Gy) splenocytes were used to stimulate antigen-specific T-cell responses from sorted T lymphocytes at a 5:1 ratio. IFNγ production was measured via ELISpot assay (R&D).

### Retroviral transduction

A retroviral expression construct (pMSGV2) encoding full-length HPV6 E6 with an NGFR tag and a plasmid encoding VSV-G were cotransfected into 293GP cells using Lipofectamine 2000. Viral supernatant (48 h) was transduced into MOC1 cells as described^19^.

### In vivo therapeutic studies

Parental MOC1 cells or MOC1 engineered to express HPV6 E6 (MOC1-E6) were transplanted via subcutaneous injection (5 × 10^6^ cells/injection) into the flanks of mice in a 50/50 Matrigel. Mice bearing 100 mm^3^ volume (day 10) tumors were vaccinated in the contralateral flank with 10^9^ viral particles twice weekly for 2 weeks. In some experiments, T-lymphocyte populations were depleted with anti-CD4 (clone GK1.5, BioXCell) or anti-CD8 (YTS 169.4) antibodies (200 µg antibody/injection).

### TIL culture

MOC1 or MOC1-E6 tumor-bearing mice were treated with two vaccinations. Forty-eight hours after the second vaccination, tumors were harvested, divided into 1 mm fragments, and cultured in RPMI 1640-based media supplemented with recombinant murine IL-2 (100 units/mL). After 7 days of culture, TIL was assessed for HPV antigen-specific responses or for their ability to kill parental MOC1 or MOC1-E6 targets via impedance analysis.

### Impedance analysis

Real-time impedance analysis was performed using the xCELLigence Real-Time Cell Analysis platform per manufacturer recommendations and as previously described^20^.

### Immunofluorescence

Paraffin-embedded tumor sections were deparaffinized, blocked, and stained with primary antibodies as described^21^. Sequential staining was achieved with anti-mouse CD4 (clone 4SM95), Opal520 (PerkinElmer), CD8 (4SM15), and Opal620 with washes performed in Amplification Plus buffer (PerkinElmer). Nuclei counterstaining was achieved with Spectral DAPI. Slides were imaged on a Vectra Polaris Imager (PerkinElmer) and analyzed with QuPath software^22^.

### Statistics

Illustrations were created using BioRender (biorender.com). All analyses were performed using GraphPad Prism v7 as previously described^20^.

### Reporting summary

Further information on research design is available in the Nature Research Reporting Summary linked to this article.

## Supplementary information

Supplementary Information

Reporting Summary

## Data Availability

The authors declare that all data supporting the findings of this study are available within the paper and its Supplementary Information files.
