# Supplementary material for: Preclinical study of a novel therapeutic vaccine for recurrent respiratory papillomatosis
Source: NPJ Vaccines. 2021 Jun 18;6:86. doi: 10.1038/s41541-021-00348-x (PMC8213691; doi:10.1038/s41541-021-00348-x)
Supplement: Supplementary file 1 — Supplementary Information [file 41541_2021_348_MOESM1_ESM.pdf]

Supplemental Figures

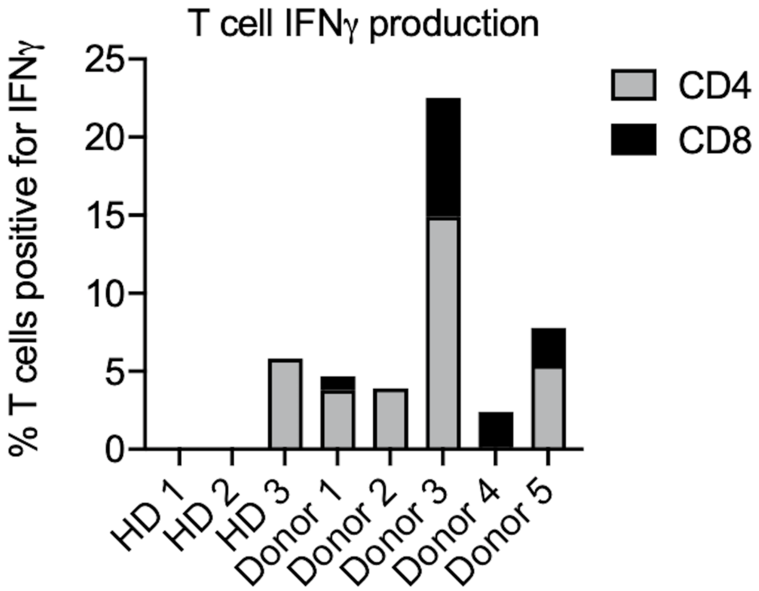

Supplementary Figure 1 – PRGN-2012 induced IFNγ production in both CD8+ and CD4+ T lymphocytes

Quantification of percentage of T lymphocytes positive for IFNγ assessed via intracellular flow cytometry following in vitro stimulation as in Figure 1.

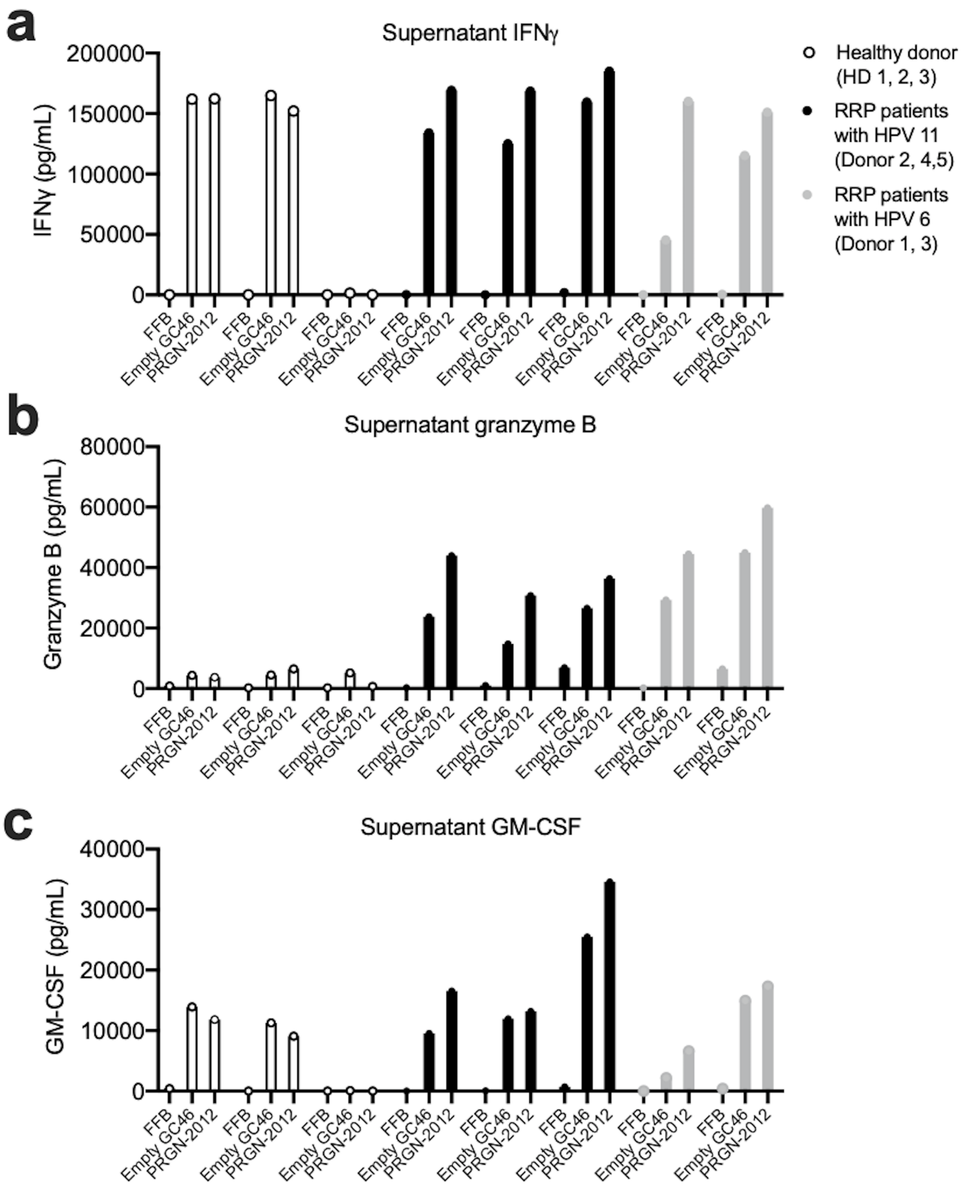

Supplementary Figure 2 – PRGN-2012 induced both antigen-specific and antigen-independent inflammation

Un-normalized supernatant quantification of (**A**) IFNγ, (**B**) granzyme B and (**C**) GM-CSF from co-cultures assessing HPV antigen-specific responses in T lymphocytes isolated from the blood of patients with HPV 11-driven RRP (n = 3, black bars), HPV 6-driven RRP (n = 2, grey bars) or healthy donors (n= 3, open bars) following two rounds of *in vitro* simulation with PRGN-2012 or empty GC46.

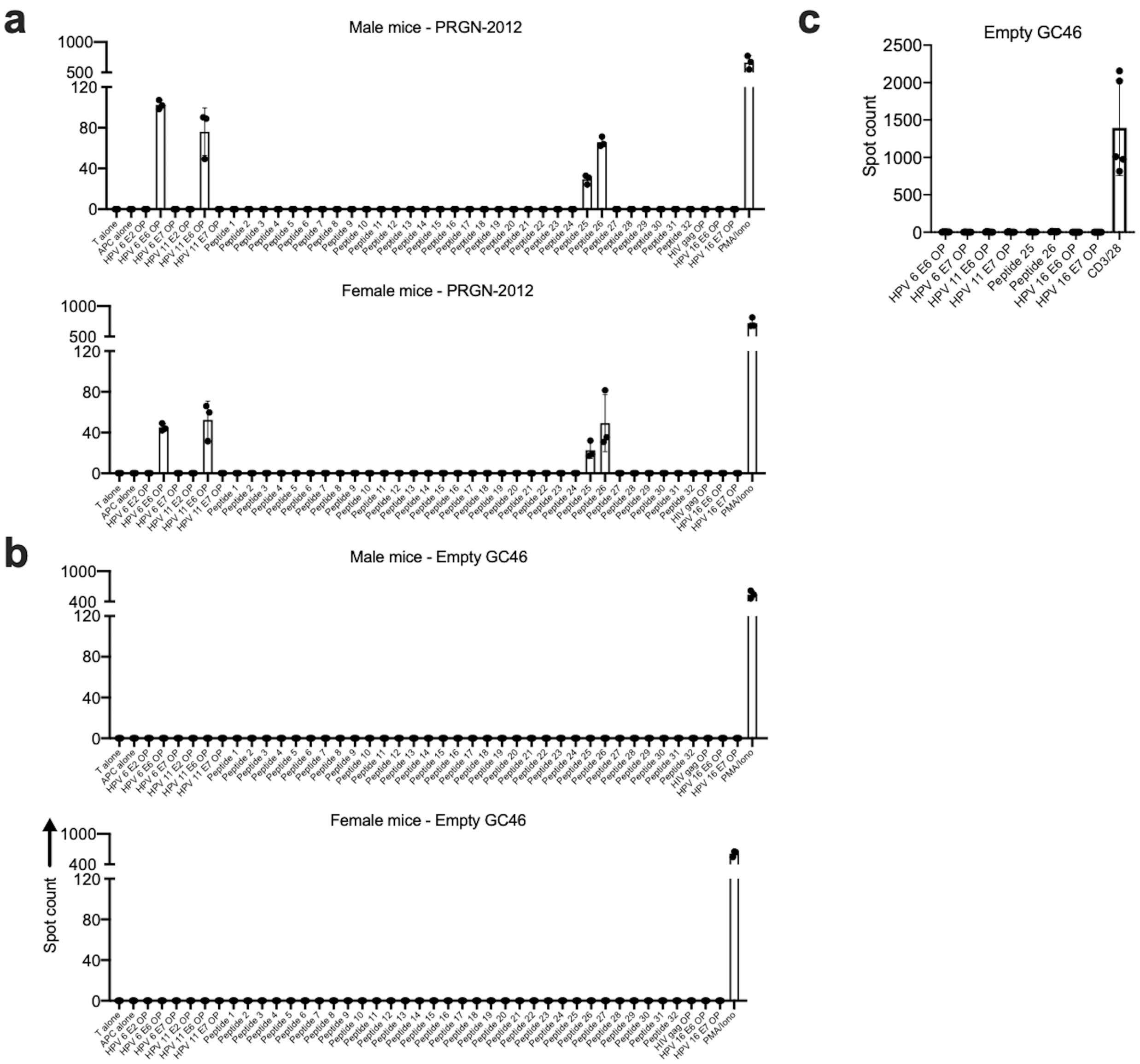

Supplementary Figure 3 – Induction of HPV antigen-specific immunity following vaccination in male and female mice

Quantification of IFNγ spots in male and female mice (n = 3/group) vaccinated with (**A**) PRGN-2012 or (**B**) empty GC46.

C, quantification of IFNγ spots in female mice vaccinated with empty GC46 in an independent experiment.

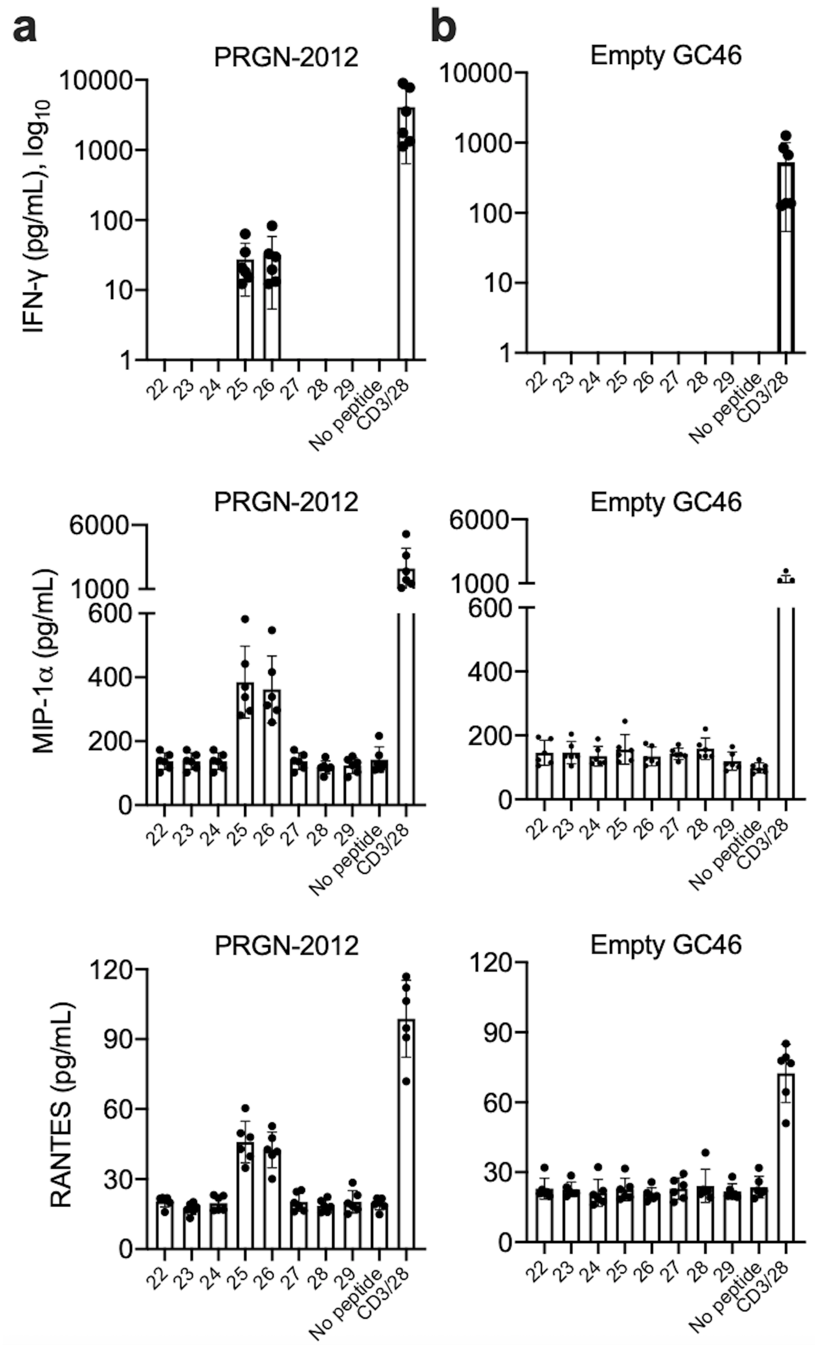

Supplementary Figure 4 – PRGN-2012 induced antigen-specific inflammatory cytokine production in vaccinated mice

Quantification of supernatant IFNγ, MIP-1a and RANTES from co-cultures assessing peptide-specific responses in splenic T lymphocytes from mice (n = 6/group) treated with (**A**) PRGN-2012 or (**B**) empty GC46 vector.

Supplementary Table I. HLA class I haplotypes of RRP patients whose blood products were used for in vitro stimulation with PRGN-2012.

Identification	HPV serotype	HLA-A	HLA-B	HLA-C
Donor 1	6	*01/*02	*08/*37	*06/*07
Donor 2	11	*01/*32	*08/*14	*07/*08
Donor 3	6	*03/*11	*14/*18	*05/*08
Donor 4	11	*01/*01	*08/*08	*07/*07
Donor 5	11	*01/*24	*08/*39	*07/*07
